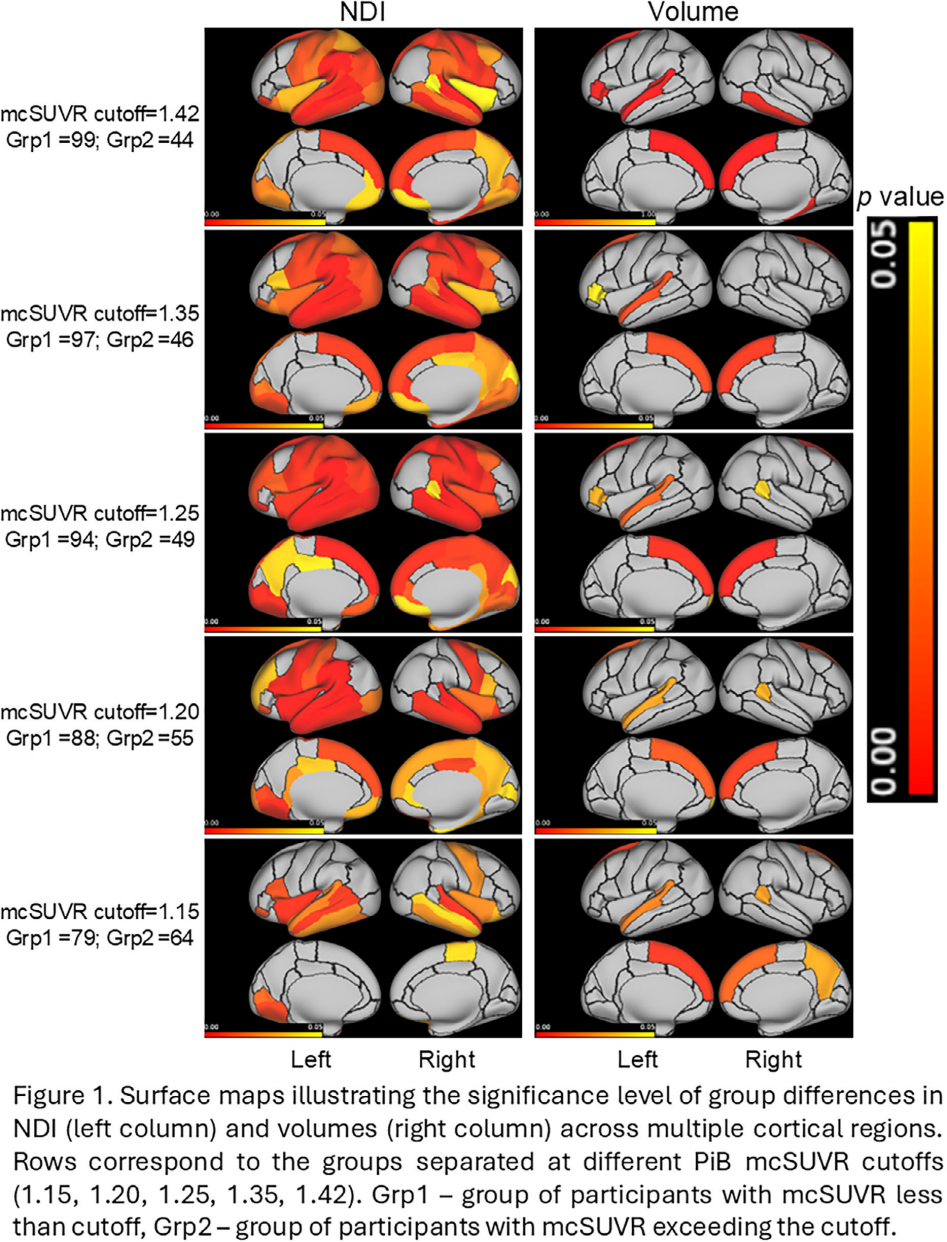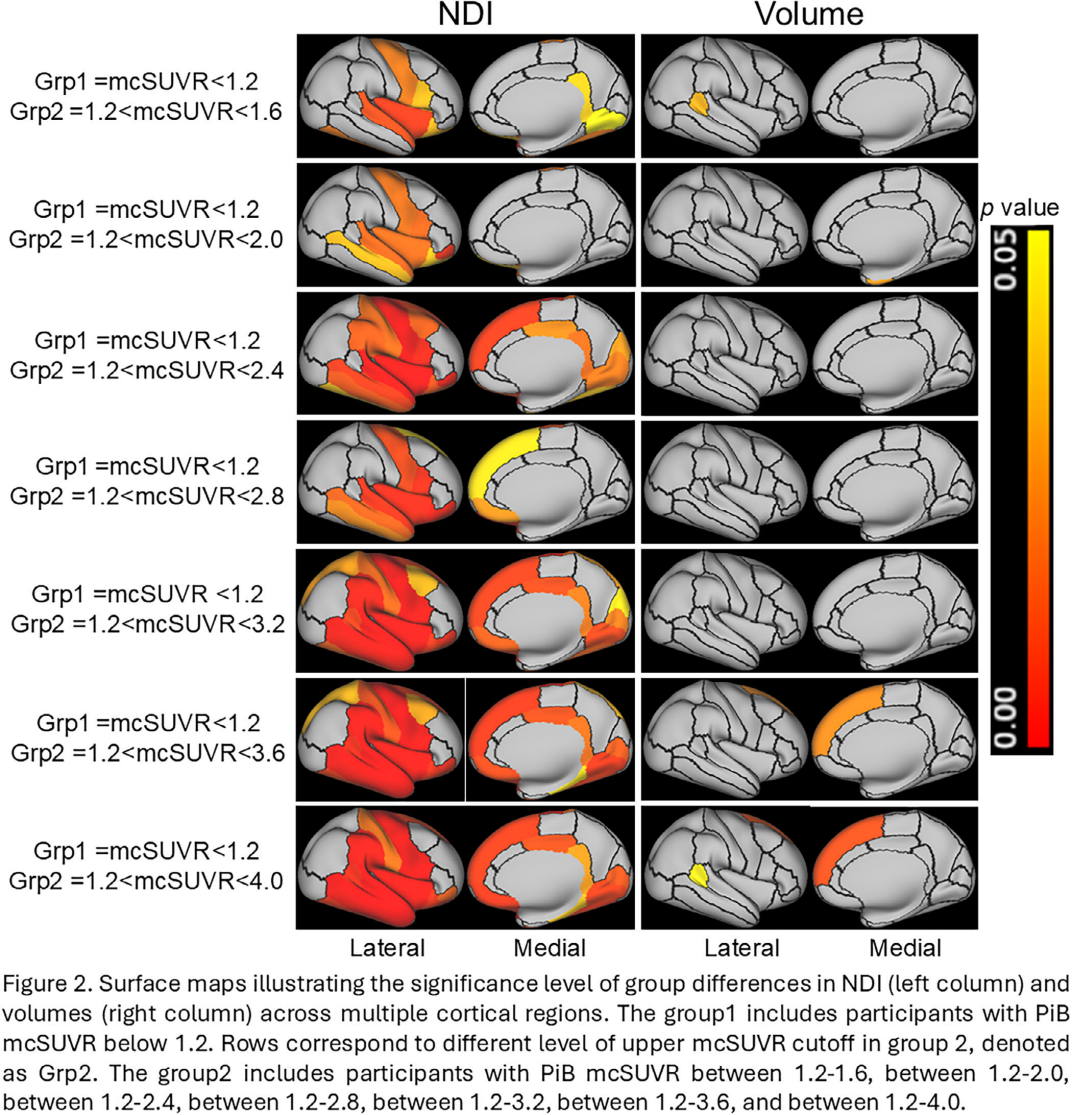# Pre‐atrophic Neurodegeneration: A Novel MRI‐Based Biomarker for Early Neuronal Injury Coincident with Early Amyloid Accumulation

**DOI:** 10.1002/alz70861_108277

**Published:** 2025-12-23

**Authors:** Satya V.V.N. Kothapalli, Tammie L.S. Benzinger, Randall J. Bateman, Melody Li, Cihat Eldeniz, John C. Morris, Dmitriy A. Yablonskiy

**Affiliations:** ^1^ Washington University School of Medicine in St. Louis, St. Louis, MO USA; ^2^ Hope Center for Neurological Disorders, Washington University School of Medicine, St. Louis, MO USA; ^3^ Knight Alzheimer Disease Research Center, Washington University School of Medicine, St. Louis, MO USA

## Abstract

**Background:**

Early identification of neuropathological changes in Alzheimer's disease (AD) is essential for the development and application of disease‐modifying therapies. Currently, brain accumulation of β‐amyloid (Aβ) and/or CSF amyloid level reduction are considered among the earliest AD biomarkers, and both are indirectly associated with brain neurodegeneration. Direct methods identifying neurodegeneration are usually based on MRI measurements of brain volume reduction (atrophy). Herein we use a quantitative Gradient‐Recalled‐Echo (qGRE) MRI method, providing a sensitive biomarker (Neuronal Density Index, NDI) for quantifying the neuronal cell damage (pre‐atrophic neurodegeneration) before it can be detected by atrophy measurements. While atrophy appears at late stages of amyloidosis, our results show that NDI can serve as a biomarker of pre‐atrophic neurodegeneration coincident with early amyloidosis.

**Methods:**

143 participants, ages between 48 and 88 (69.5±7.1; 85 females; 58 males), recruited through the Knight ADRC and SEABIRD study, provided informed consent. qGRE and PET amyloid ^11^C‐PiB images were acquired concurrently. For each participant, amyloidosis level was identified by mean cortical standard uptake ratio (mcSUVr), while NDI was quantitated in all cortical FreeSurfer‐defined brain regions. Group differences between participants’ NDIs were established for a range of mcSUVR cutoff levels.

**Results:**

A standard mcSUVR cutoff of 1.42 is usually used to separate participants into Aβ(‐) and Aβ(+) groups. Results in Figure 1 show dramatic pre‐atrophic neurodegeneration in Aβ(+) group with NDI significantly decreased in multiple brain regions, while significant volume changes were observed only in frontal and insular regions. Furthermore, the NDI exhibited significant group differences in multiple brain regions even when mcSUVR cut‐off threshold was reduced to 1.2. Importantly, NDI exhibited significant changes in medial temporal lobe even when comparing groups with mcSUVr < 1.2 and mcSUVR between 1.2 and 1.6 (Figure 2). In contrast, volume measurements did not show significant differences until participants with mcSUVR greater than 3.4 were included in the analysis.

**Conclusion:**

qGRE‐based NDI provides in‐vivo microstructural brain neurodegeneration distribution in early amyloid pathology where atrophy is not detectable. Although the NDI parameter reflects neuronal cell density, presence of inflammation can also reduce the NDI value. Thus, further investigations are required to understand the microstructural nature of pre‐atrophic neurodegeneration.